# A Solution to the Measurement Problem in the Idiographic Approach Using Computer Adaptive Practicing

**DOI:** 10.3390/jintelligence6010014

**Published:** 2018-03-02

**Authors:** Abe D. Hofman, Brenda R. J. Jansen, Susanne M. M. de Mooij, Claire E. Stevenson, Han L. J. van der Maas

**Affiliations:** 1 Department of Psychological Methods, University of Amsterdam, 1018 WS Amsterdam, The Netherlands; c.e.stevenson@uva.nl (C.E.S.); h.l.j.vandermaas@uva.nl (H.L.J.v.d.M.); 2 Department of Developmental Psychology, University of Amsterdam, 1018 WS Amsterdam, The Netherlands; b.r.j.jansen@uva.nl; 3 Department of Psychological Sciences, Birkbeck, University of London, London WC1E 7HX, UK; sdemoo01@mail.bbk.ac.uk

**Keywords:** idiographic approach, computerized adaptive practicing, intraindividual variation, cognitive development, mathematics

## Abstract

Molenaar’s manifesto on psychology as idiographic science (Molenaar, 2004) brought the N=1 times series perspective firmly to the attention of developmental scientists. The rich intraindividual variation in complex developmental processes requires the study of these processes at the level of the individual. Yet, the idiographic approach is all but easy in practical research. One major limitation is the collection of short interval times series of high quality data on developmental processes. In this paper, we present a novel measurement approach to this problem. We developed an online practice and monitoring system which is now used by thousands of Dutch primary school children on a daily or weekly basis, providing a new window on cognitive development. We will introduce the origin of this new instrument, called Math Garden, explain its setup, and present and discuss ways to analyze children’s individual developmental pathways.

## 1. Introduction

The human cognitive system, and especially its development in the first 10 years, is inconceivably complex. Of all complex systems that are now studied in science, such as ecosystems, the climate, the immune system, and stock markets, the developing human cognitive system is by far the most challenging [1]. One common element in the study of the dynamic complex systems is the focus on high quality and highly frequent measurements. Such data, collected within an individual, are required to study individual development in detail and to answer fundamental questions on cognitive development and learning [2,3]. This idiographic approach is also important for educational approaches based on personalized learning. To adapt education to the individual, highly frequent samples of reliable data on learning processes are required. Within developmental psychology, Siegler [4] stressed the importance of frequent sampling to collect longitudinal data (the microgenetic method). The idiographic and microgenetic approach share many ideas and concepts, although the latter does not necessarily imply using only intra-individual data. The microgenetic method is defined by three key components: (1) observations span the full period of the developmental process; (2) the density of observations is high, relative to the rate of change; (3) and the analysis aims to infer the process that gives rise to both quantitative and qualitative changes [4].

**Figure 1:**
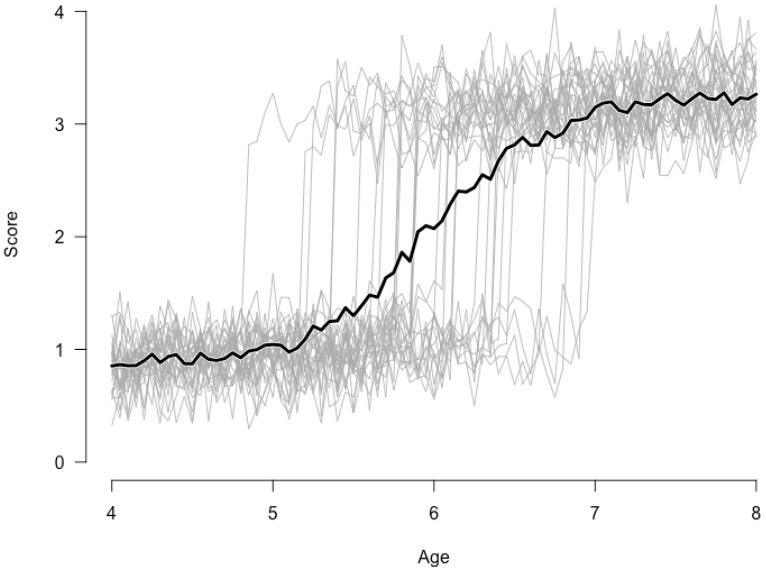
The (misleading) average of multiple individual curves.

Figure 1 provides an illustration of the importance of idiographic analysis for qualitative changes. Here we consider the case of a sudden transition in some developmental process for 30 simulated children. Suppose this process can be described by a switching regression model per individual, where each child goes through a similar sudden change in development. Up until some age (the precise age differs per subject and is sampled from a normal distribution with mean M=6 and standard deviation SD=0.5) the developmental curve is based on a low intercept (M=0.5;SD=0.1). After this age the intercept changes abruptly (M=2.5;SD=0.1). Slopes before and after this sudden jump are rather flat (M=0.1;SD=0.02). Some measurement error is added (M=0;SD=0.2). Figure 1 clearly shows within subject sudden changes for all 30 children. The darker line, however, representing the group’s average performance at each point in time, suggests a more continuous developmental process. Such between subject data would have been collected in a typical cross-sectional study. Although, in this case a more advanced between subject analysis—a mixture approach for instance [5]—would reveal the discontinuous character of the developmental process.

Idiographic analyses are thus essential in revealing the dynamics of development. However, it has been very difficult to develop reliable and valid measurement instruments for cognitive development that can be used for the full period of developmental processes. It is even more difficult to construct these instruments in such a way that they can be used in high frequent measurements, say once a week or once a day. Moreover, even if such tests were available, recruiting subjects would be very hard. Most schools would not agree to daily or weekly test sessions for say an hour per session for long periods of time.

Over the last 10 years we developed a new system meeting both scientific and educational objectives, that solves these problems. With this system, the main application being Math Garden (in Dutch: Rekentuin) [6,7] we have collected high-frequency time series of thousands of subjects over multiple years in many scholastic and cognitive domains. We first describe and explain the basic technology underlying Math Garden. Second, we present examples of analysis of intraindividual data. Third, we discuss some limitations of our approach.

### 1.1. Computer Adaptive Practice

The inspiration for the system that solves an important number of the measurement problems of the idiographic approach to development was the observation that children solve many practice exercises in math and language almost daily. Actually, school children spend about 50% of their time on reading, writing, and arithmetic [8]. A substantial part of this time is devoted to practice exercises. If we could obtain the data of these practice exercises, a new measurement system could be in reach. However, the diversity of practice materials, which are not setup as measurement instruments, makes such an approach impractical.

To acquire measurements that are scientifically useful, we developed a new exercise approach for education that is both educationally and scientifically valuable. Understanding this approach requires some knowledge of modern test theory. Modern test theory provides techniques for educational measurement, among them computerized adaptive testing [9]. Modern test theory involves various item response models that specify the probability of a correct answer given characteristics of the person and the items, in the simplest case the ability of a person and the difficulty of the item. The person ability and item difficulty are expressed on the same latent scale. In the most basic model, the Rasch model [10], the probability is 0.5 when the ability of the person equals the item difficulty. If a person’s ability is much higher the probability approaches 1, and vice versa 0 Person abilities and item difficulties are related to the more traditional test indices such as sum scores for persons and p-values for items. However, there are important advantages of the Rasch model compared to the more traditional test indices. The main advantage relevant for our approach concerns adaptive testing. In computerized adaptive testing (CAT), people do not have to solve all items in a test; they are only presented with a limited set of informative items, depending on their successes and failures on earlier items. More difficult items follow successes and easier items follow failures. Tests are much shorter since uninformative (too easy or too difficult) items are not administered, which results in a quicker estimation of a person’s ability. In CAT the item bank consists of hundreds or even thousands of items.

Computer adaptive testing is however not directly applicable to computer adaptive practice. In order to use CAT in an educational practice system, we had two problems to solve. The first problem concerns the pretesting of items. Note that CAT assumes that all item difficulties are known. This means that all items in the item bank should be pretested on many subjects before one can start CAT. Since our computer adaptive practice systems (Math Garden and Language Sea) consist of about 40 games, with in total more than 50.000 items, pretesting is impractical.

The second problem is more technical but important. In CAT based on the Rasch model the most informative subsequent item is an item for which the expected probability correct is about 0.5. This implies that subjects fail on about 50% of the items. In an educational practice system this is unacceptable and unethical. Although selection of easier items is possible in CAT, the speed of convergence in estimating a person’s ability drops considerably [11]. Clearly, if a large majority of answers is correct we do not learn much about a person’s ability.

We solved the problem of pretesting by using an online estimation method that originates from the world of chess. In the Elo rating system, ratings (abilities) of players are updated after each game by a simple update formula [12]. In this update formula, the difference between the expected outcome computed from the ratings of the players prior to the game and the actual outcome of the chess game is used to compute the new ratings. The advantage of Elo’s dynamic estimation method is that it does not depend on pretesting, it can start with arbitrary initial ratings. We can set all players’ ratings to 1500, let players play chess, update ratings and the ratings will converge after some time to values that accurately represent (differences in) playing strength (in chess, ratings vary between 1000 and 3000).^1^

In Math Garden we apply the same system with some important modifications. First, people play against items (and not other people). A person’s ability (ratings) increases when they solve the item correctly, and decreases when they fail, and vice versa for the item difficulties. Mathematical details of our adaptation of the Elo system can be found in [6] and especially [13].

We solved the second problem by integrating response times into the scoring of responses. As mentioned, correct performance on very easy items is not very informative about a person’s ability, however speed of responding is [14]. Yet, children may have different speed accuracy trade-off strategies; some may favour accuracy over speed while others favour speed over accuracy. For instance, the ability of children who play cautiously may be underestimated. To minimize the influence of differences in speed accuracy trade-off we developed an explicit scoring rule for the weighing of accuracy and speed. This scoring rule, called item residual time rule, weighs accuracy (+1,−1) with the remaining time for an item [13]. Given a time limit of, for instance 20 s, a correct answer in 8 s gives a score of +12, whereas an error in 5 s yields a score of −15. Thus fast guessing is discouraged. Maris and van der Maas [13] show that this scoring rule implies the two parameter logistic item response model [15] with the item time limit as a discrimination parameter. The integration of response times into the response scores enables us to present children with items for which they have an average probability of 0.75 of solving the items correctly. Given a person’s current ability estimates a set of items is selected with a 0.75 expected probability correct. From this set a random item is selected that has not been played recently by the child [6].

This scoring rule forms the basis of the extended Elo system used in Math Garden and other applications in the system. In the games, the scoring rule is represented by virtual coins, equal to the time in seconds available for the item. Each second a coin disappears. In case of a correct answer, the child wins the remaining coins. In case of an error, the remaining coins are subtracted from the total coins won. In this way, the scoring rule is understandable for young children and adds a gamifying element to the task, keeping the children motivated. Children can buy virtual prizes, such as flags and trophies, using the collected coins. Because the games are adapted to player ability level, the coins and prizes they win are independent of ability and only depend on the frequency of playing. Hence, the possibility to win by mainly playing, also encourages weak players to practice a lot. If children do not want to answer an item for any reason, they can click a question mark, which replaces the item with another item and no coins are won or lost.

### 1.2. Math Garden

These innovations are applied in Math Garden, and later other learning platform websites for children such as Language Sea, a language learning program; Words and Birds, a learning platform for learning English as a secondary language and Type Garden, an adaptive e-learning environment that teaches children to touch type [16]. In Math Garden each plant represents a math game that grows as ability increases. Currently Math Garden contains games for many different mathematical skills, such as basic arithmetic operations, but also counting, series, fractions, and clock reading. Other games concern more general cognitive abilities, such as working memory, deductive reasoning, and perceptual abilities.

These online games allow children to practice intensely at their own ability level with direct feedback, two important requirements for deliberate practice [17]. Teachers are provided with learning analytics for each individual child. Each online learning platform is a self-organizing learning tool that does not require extra effort from teachers. Note that these websites are not teaching methods. They take over practicing and monitoring tasks, but not the instruction (although some games give intelligent feedback).

Math Garden and Language Sea are popular in the Netherlands. About two thousand schools have bought subscriptions for some classes or the whole school. Schools agree with the use of anonymized data for scientific research. At the end of 2017, more than 200,000 primary school children in the Netherlands use these websites regularly. On weekdays about 1.5 million item responses are collected per day. About 25% of the response are collected after school hours.

## 2. Scientific Analysis

This type of ‘big’ data is extremely promising. It contains highly frequent ‘modern test theory’ measurements of the development of numerous abilities from children of a wide range of ages. In a sense, it provides a new window on intellectual development.

On the other hand, new problems arise. First there is the issue of user privacy. It is crucial to de-identify the data carefully. Second, data are collected with a variety of devices, at home or at school, children might receive help, use each other’s accounts, etc. Third, big data analyses are often exploratory with all of the associated risks, such as accidental relations between variables. Finally, in the analysis of Math Garden data many, often rather arbitrary, choices have to be made about data selection and handling of missing data. Our general approach is to check the robustness of results with different data selections and different types of analyses.

In the last 5 years about 25 papers using Math Garden and Language Sea data have been published (for example, see [18,19,20,21,22,23,24,25,26,27,28,29,30,31,32]. For instance the development of the ability to solve three-term arithmetic expressions (such as 3+4×6) was studied [33]. The question was whether the development in the domain of mathematics involves a shift from non-formal mechanisms to formal rules and axioms or, alternatively, involves an increase in reliance on non-formal mechanisms. Math Garden data from about 50,000 children were analyzed. So-called foil errors were more common for problems in which formally lower-priority sub-expressions were spaced close together. These effects increased with the children’s grade level, suggesting that these mechanisms do not vanish but actually become more important over development.

A second example concerns the inversion error when writing down number words in symbolic notation (also called “transcoding”) [34]. In Dutch and many other languages, transcoding is complicated by decade-unit inversion: 24, for instance, is pronounced as ‘four-and-twenty’. It was shown, using Math Garden data, that the incidence of these errors declined but did not disappear in later elementary school. In addition, transcoding ability mediated the relationship between visuospatial working memory and mathematics performance, a strong effect that declined with age.

Many of these papers center on interindividual differences and item effects. In this paper, we will focus on the analysis of intraindividual data.

### 2.1. Intraindividual Analysis

Mathematical proficiency is essential for functioning in today’s society. Higher proficiency levels are associated with higher levels of employment [35,36] and are, for example, necessary for making well-informed choices about health and health care [37]. Despite the importance of mathematics, relatively little is known about the intraindividual development of mathematical abilities [38].

**Figure 2:**
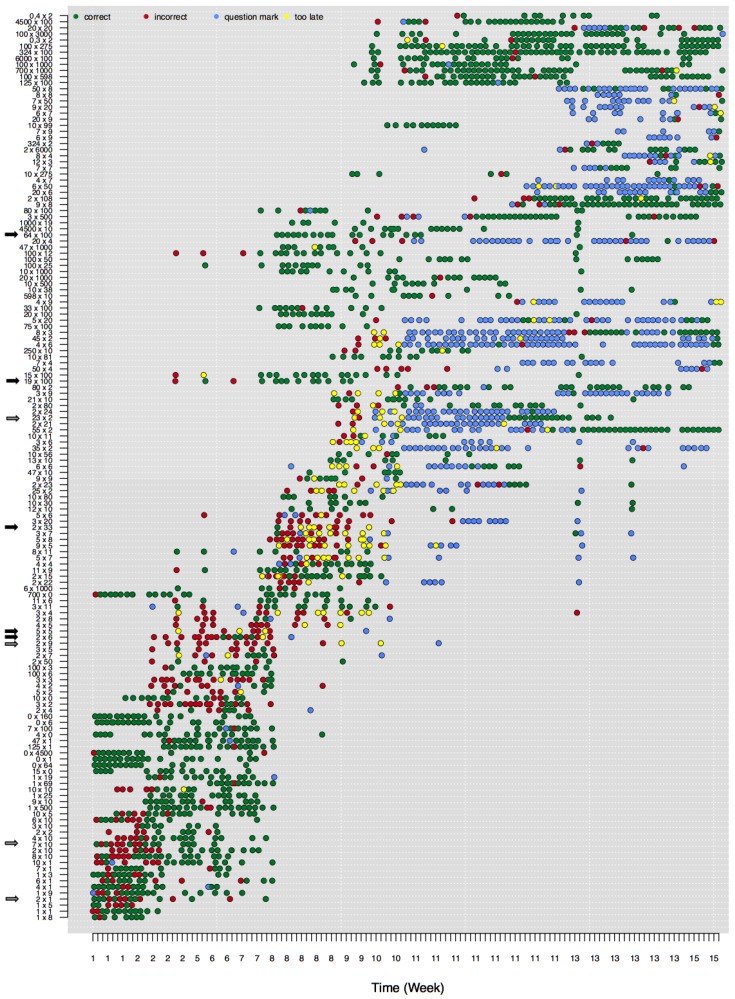
One player’s development in learning to solve multiplication problems correctly. The colors refer to correct (green), incorrect (red), question mark (blue) or responses that were too late (yellow). The minimum number of responses for each time-series was 5. The items are sorted by item difficulty (low = easy and high = difficult). Plots for other players, providing different patterns, are available on www.abehofman.com/papers. The arrows along the Y axis indicate the items that are further described in the text (black arrows illustrate different response patterns; grey arrows indicate different trajectories of mastery).

Here we explore how to visualize, describe, and analyze data at the detailed level of an individual’s responses to single math problems. In this specific illustration, a subset of Math Garden’s addition and multiplication data is used. These data stem from children who have visited Math Garden almost daily and who played frequently for prolonged periods. The data consists of a large set of person-by-item time-series: time-series of responses of a single child to a single item. The large amount of data on learning of individual children that is unlocked by these time-series is illustrated in Figure 2. Figure 2 shows the development of an individual child’s multiplication skills from weeks 1 to 15 (horizontal axis). The vertical axis represents the difficulty of the attempted multiplication problems. A dot in the graph shows that the child has attempted to solve this problem, whereas the color shows the accuracy of the response. Figure 2 shows that the difficulty of the attempted problems increases in time.

The child whose data are plotted in Figure 2 starts, in week 1, with correct and some incorrect responses to easy multiplication items. Due to the CAT routine, only a subset of available items are presented at each time-point. Around week 2, this child seems to master items with a times 10 operator. As a consequence the child’s ability estimate increases (not represented in Figure 2) and more difficult items are presented. Responses to the more difficult set of items (e.g., 2×6 and 5×5) are more often incorrect, as is predicted from the measurement model. Around weeks 8, 9, and 10 even more difficult items are presented (e.g., 2×33 and 19×100), but now the child consistently succeeds at quite difficult problems but fails at easier problems, indicating some model misfit. This misfit is especially prominent for the long series of only correct responses to more difficult items (e.g., 64×100 and 100×12). The most difficult items (at the top of the figure) are almost always solved correctly, while the easier items—including some items that belong to the standard multiplication tables—are still solved incorrectly.

Furthermore, the trajectory of mastery differs remarkably between items. Whereas some items seem to be learned slowly (e.g., 7×10), other items are mastered suddenly, from one attempt to the next (e.g., 23×2). For other items it is even unclear whether they are mastered at all, since the player continues to switch between correct and incorrect responses (e.g., 2×1). Also, other items are no longer presented to the child even though the last responses to the items were incorrect (e.g., 2×9).

The visual inspection of these figures highlights many interesting patterns. However, the large number of users in these systems, combined with the fast rate with which responses are collected, makes it impossible to inspect these plots for all users. Hence, learning analytics are needed to characterize different patterns of learning, to highlight users who show interesting (deviating) developmental patterns, and in the end to use such analytics to provide teachers and children with person-specific support on their learning process.

## 3. Study 1: Learning Analytics

We developed and investigated learning analytics to characterize different learning patterns. These analytics are aimed to describe per item: (1) whether the child learned the item; (2) the learning pattern; (3) the stability and variability of responses over time. We focus on learning analytics that are feasible in a big data setting. The learning analytics should thus be fairly simple and easy to compute.

For the first analysis, we collected responses of frequent players from the addition and multiplication games between 1 September 2013 and 1 July 2017. To this end, we first selected players with more than 1500 responses (*N* is 5339 and 4714, respectively). We only used data of subjects that played at the most difficult level (with an expected probability correct of 0.6, see [25]). In both games so-called mirror items exist. Mirror items are items that only differ in the order of the operands (e.g., 1+2 and 2+1). Since these mirror items are closely related, responses to both mirrors were combined within a single time-series. In a second step, we omitted all responses to (mirror) items with less than five observations and only included players when they provided at least 250 responses to 25 different items.

The data selection procedure resulted in a data set of 2287 (mean age = 8.12, SD = 2.01) and 2867 players (mean age = 8.89, SD = 1.73) for the domains of addition and multiplication respectively. The data included in total 1,040,321 and 2,090,822 responses to 1169 and 740 mirror items.

To characterize individual learning curves, a number of candidate learning statistics were computed for each person-by-item time-series. These were the following:

1. Response probabilities of the last two responses.
2. Transition probability matrix of correct and incorrect responses.
3. Coefficients of a logistic regression model.

These statistics were computed for correct and incorrect responses only, excluding question mark and late responses. The percentage of correct responses in the last two responses informs us whether users are able to answer an item correctly at the end of a time-series. The transition probability matrix is a 2 by 2 transition matrix, where the probability of switching from an incorrect to a correct response and the probability of remaining at a correct response are particularly informative. Since the remaining two probabilities are complementary, these are redundant and will be omitted. The transition matrix indicates persistence from incorrect to correct responses. Parameters of the logistic regression provide information on the person-by-item learning curve. The logistic function is:

(1) $$P\left(x=1|t\right)=1/\left(1+e^{-\beta_{0}+\beta_{1}x_{t}}\right)$$

where $\beta_{0}$ is the intercept and $\beta_{1}$ is the slope (steepness) of the learning curve. We used the position in the time-series as the explanatory variable ($x_{t}$). The slope of the learning curve reflects the learning speed. A flat curve indicates that an item was already mastered at the start of data collection, or that an item was not mastered during data collection, the value of the intercept ($\beta_{0}$) indicates when learning occurred.

We use Bayesian logistic regression instead of regular logistic regression, because the latter cannot handle complete separation [39]. Complete separation occurs when a developmental trajectory involves a perfect step-like function between different states [3]. The models were fit using the arm package [40] in *R* [41] using default priors. BIC differences were calculated between models with and without a slope parameter to compare the contribution of this parameter to model fit.

### Results

In the addition data set 35% of the series ended with two correct responses. This percentage is close to the implied probability by the measurement model of 0.36 $\left(0.6^{2}\right)$.^2^ For the multiplication data set 47% of the series ended with two correct responses, higher than the expected probability of 0.36.

**Figure 3:**
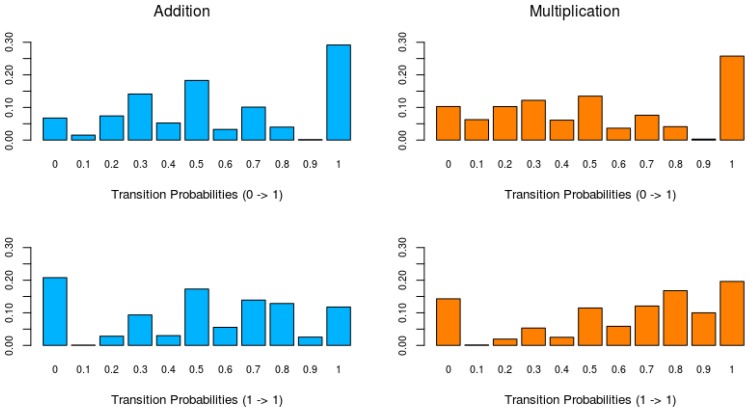
The distribution of the transition probabilities of switching from incorrect to correct responses (**top**) and remaining at a correct response (**bottom**) for addition (**left**) and multiplication (**right**) for all collected time-series. For, example, the transition (learning) probability (0→1) of 0.7 indicates that 70% of incorrect responses are immediately followed by a correct response. The bar at 0.7 in the upper-left panel indicates that this is the case in about 10% of all the collected series in the addition game.

Figure 3 shows histograms of the switching probabilities between an incorrect (0) and correct response (1) (learning probability), indicating learning, and the probabilities of remaining at a correct response, for each domain. The upper histograms of Figure 3 show that for the learning probabilities of both domains there is a clear peak at one (bar on the right), indicating that in about 30% of the addition series and 25% of the multiplication series the learning probability is one. This probability of one indicates that an incorrect response is followed by a correct response. The lower histograms of Figure 3 show the probabilities of remaining at a correct response. For both addition and multiplication, there is a large peak at zero, indicating that in 20% of the series for addition and 15% of the series for multiplication a correct response is always followed by an incorrect response. The low frequency of remaining in the learned state implies that the switch from incorrect to correct responses is not very stable. Furthermore, a comparison of the probabilities of remaining in the learned state (1→1) between the addition and multiplication data shows that for addition these probabilities are lower than for multiplication.

**Figure 4:**
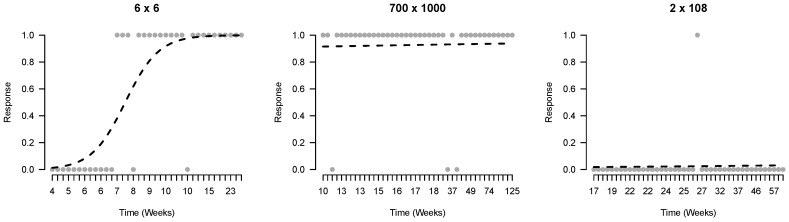
An example of three different developmental patterns of responses to different items by the same player. The left panel shows a time-series with a clear increase in the probability of a correct response. The middle and right panel respectively show a series of a previously learned item and a series that indicates no learning.

Third, we investigate the evidence for learning in the time-series by fitting learning curves with logistic regression models. To explore the fit of these models to the observed time-series we plotted the observed and predicted responses of three series of the same player, shown in Figure 4.

**Figure 5:**
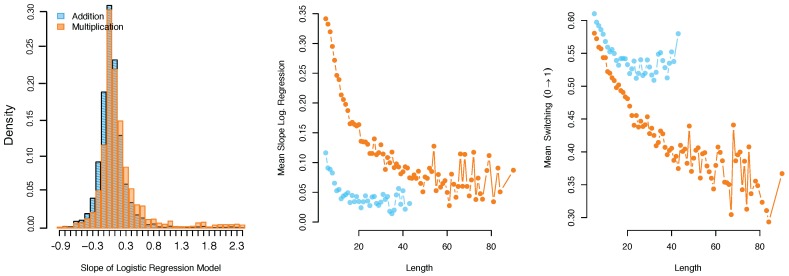
The distribution of the estimated slope parameters, i.e., indicator of learning speed, for both data sets (**left**-panel), and the relation between the length of the series and learning speed (**middle**-panel) and the probability of switching from an incorrect to a correct response (**right**-panel).

For the addition data set 19% of the models fitted to the time-series included significant slope (i.e., learning speed) parameters (as indicated by the BIC difference between the model with only an intercept and the model with both a slope and an intercept parameter). Of these time-series that included a significant slope, 70% of the slopes were positive. For the multiplication data set 36% of the series were best described with a model including a slope parameter, and 86% of these were positive. Negative slopes occur, for instance, when children answer numerous item administrations correctly, but fail on the item once or twice at the end of the series. So for both data sets only a minority of learning curves show an increase in mastery. Yet, the slope parameters for multiplication were higher (average $\beta_{1}=0.25$) than the slope parameters for addition (average $\beta_{1}=0.08$), see also the left panel of Figure 5. Furthermore, for both data sets the time-series length was negatively correlated with slopes and learning probability in the transition matrix, indicating that learning speed was negatively related to the number of times a child spent solving an item. This negative effect can be explained by the adaptivity of the learning program. If children learn and their ability estimates increase accordingly, more difficult items will be selected. Hence, long time-series can only be collected if children do not show large changes in their ability.

For a better understanding of these learning curves, we investigated differences between children’s estimated learning speeds (i.e., slopes). First, a positive correlation was found between the average learning speed of the addition and multiplication domains (ρ(826)=0.345,p<0.001; only players with more than five time-series in both domains were included). Second, we investigated the correlations between the slopes on different items within the multiplication domain. Based on the patterns of Figure 2 and the results of [42] two different item clusters can be defined: items that belong to multiplication tables 2 through 9 (*table* items) and items with a times 10, 100 or 1000 operator (*times* 100 items). Within the multiplication domain no significant correlation was found between these two item clusters (ρ(185)=−0.003,p=0.964). We tested whether the correlation between these two sets of multiplication items is indeed lower compared to correlations based on more similar items within the domain. To this end, we used a permutation test^3^ to calculate the correlation between learning curves of two random sets of *table* and *times 100* items. The average (within cluster) correlation for *table* items was 0.471 (SD = 0.067) and for times 100 items was 0.420 (SD = 0.050). These results indicate that players who show steeper learning curves on *table* (*times 100*) items also show steeper learning curves on other *table* (*times 100*) items, but learning speed measured by the learning slopes between items sets is unrelated. These results are surprising and will be further explored in the next analyses.

To conclude, the results about learning, stability, and change show that significant learning occurs on only a small set of the time series in these Math Garden games. Although large variations in learning can be expected [43], some variability is caused by the manner of data collection in an adaptive learning environment. In this adaptive learning environment children are presented with items that match their ability, which removes the start of the learning curve (when items are still too difficult) as well as the end of the learning curve (when items have become too easy). A second explanation for the limited observation of significant learning may be possible violations of unidimensionality. This will be the topic of the next section.

## 4. Study 2: The Problematic Assumption of Unidimensionality

Math Garden allows for the massive collection of high-frequent intraindividual data. Children might display all kinds of personal developmental trajectories, which in principle can be detected and analyzed. One limitation with regard to the implementation of the Rasch model in the system is the use of a common scale of item difficulties. In the self-organizing algorithm of Math Garden children’s responses together determine the difficulty of items. This only works when item ordering is similar for all children. Basically this is the assumption of unidimensionality in IRT. One might argue that a principled idiographic measurement approach would not assume unidimensionality [44]. It is possible that each child requires his or her own measurement scale, e.g., a person specific ordering of items. This would be highly impractical of course and probably unnecessary. Fortunately, it is possible to investigate this matter empirically.

In this section, we investigate the dimensionality of the item scales using different analytics based on an item clustering approach. This clustering approach - based on an extended measurement model [45]—is aimed to classify items into subsets of related mathematical constructs. These subsets are defined by stronger (positive) correlations within the item sets and weaker (or negative) correlations with items in other sets. These sets possibly relate to different skills within a game.

In Pelánek et al. [45], different extensions of Elo models are presented. One of these extensions, called the networked model, is especially suited for estimation of item clusters. In the networked model, local and global skills are differentiated and estimated separately. The global skill is derived from all item responses whereas the local skill is derived from the responses to just a single item. Hence, the global skill can be interpreted as a general skill for the domain (e.g., addition skill or multiplication skill), where the local skills can be interpreted as skills to solve a specific item (e.g., the skills to solve 3+4). However, it is expected that local skills cluster as various items will tap into the same skill (e.g., adding small numbers). The finding that (clusters of) local item skills are necessary to describe the abilities of children, next to a global skill, would actually be a violation of the assumption of unidimensionality.

For the estimation of the networked model, we selected the responses (accuracies) of the 200 most played items of players who completed at least 20 sessions of 15 responses between 1 September 2014 and 1 June 2017 for both the addition and the multiplication game. This resulted in 5144 users for the addition game (mean age = 7.17, SD = 1.20) and 8,180 users (mean age = 8.92, SD = 1.31) for the multiplication game. These users provided in total 2,708,027 and 4,557,333 responses for the addition and the multiplication game, respectively. In a first step, the correlation matrix of the local skills was inspected. In a second step the clustering of local item skills was explored using a hierarchical clustering algorithm. To interpret the results of the empirical data, we compared them to results of simulated data. For this simulation we generated responses with an unidimensional IRT model. In the generated data no local skills were present. Hence, when fitting the networked model to the generated data, we expected that extending the model by estimating local skills would merely result in capturing random fluctuations (error), and the correlation matrix of the local skills would be centered around zero with no specific patterns between items.

### Results

**Figure 6:**
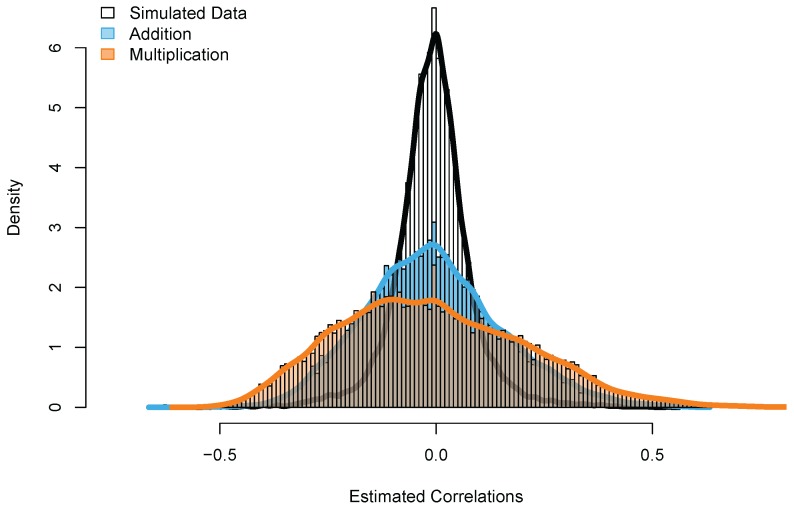
The distributions of the estimated correlations between the local skills in the addition, multiplication and simulated data.

As shown in Figure 6, the estimated correlations strongly differ between the simulated data set and the two empirical data sets. Whereas the estimated correlations for the simulated data set are around zero (as expected), the correlations of the empirical data sets show much more variation, as indicated by the large tails of both distributions in Figure 6.

**Figure 7:**
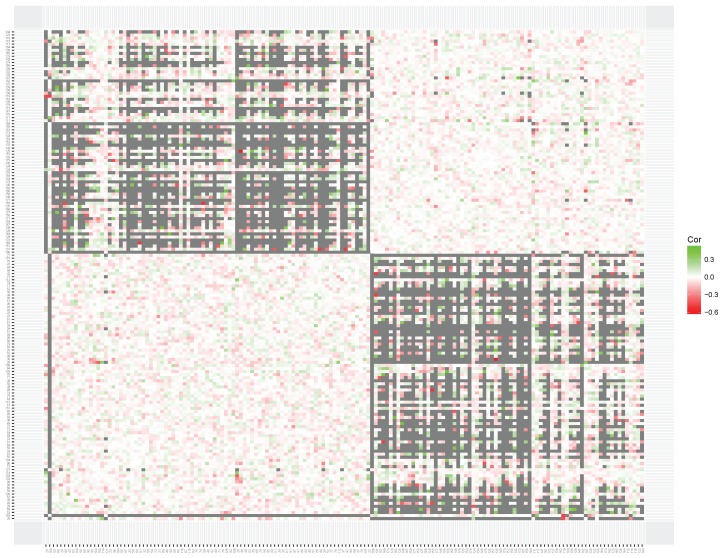
A heatmap based on the correlation matrix of the local skills estimated on a simulated data set based on the unidimensional model. Gray squares indicate missing values in the correlation matrix, resulting from adaptive item selection. The figure can also be found on www.abehofman.com/papers allowing for more detailed inspection.

**Figure 8:**
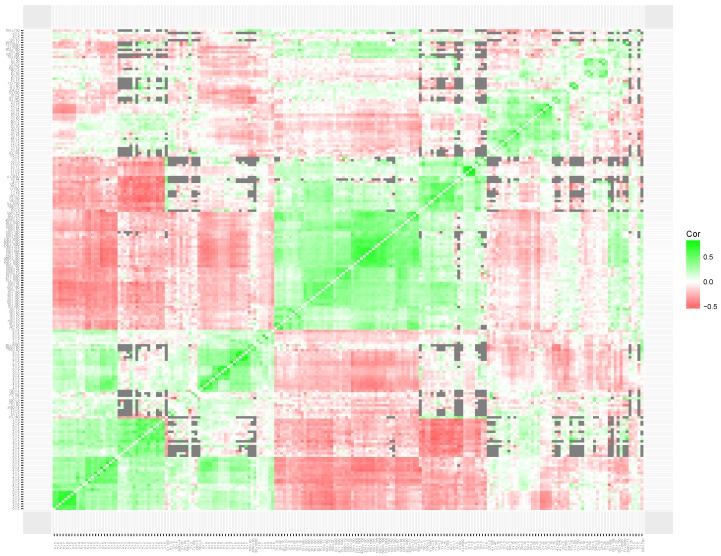
A heatmap based on the correlations matrix of the local skills of 200 multiplication items. Gray squares indicate missing values in the correlation matrix, resulting from the adaptive item selection. The figure can also be found on www.abehofman.com/papers allowing for more detailed inspection.

**Figure 9:**
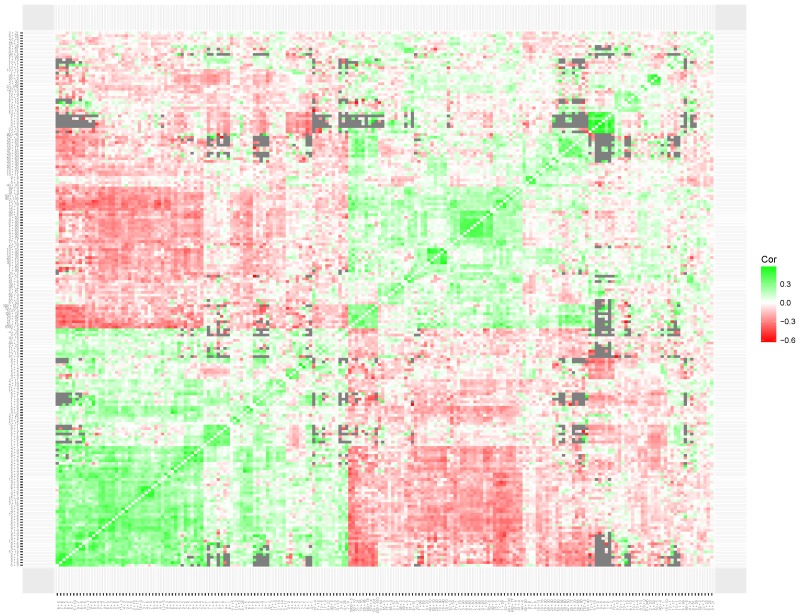
A heatmap based on the correlations matrix of the local user abilities of 200 addition items. Gray squares indicate missing values in the correlation matrix. See www.abehofman.com/papers for a downloadable version.

To interpret the results of the clustering of local item skills, we produced heatmaps for the simulated data set (Figure 7) and the empirical data sets (multiplication in Figure 8; addition in Figure 9). The heatmap based on the correlation matrix of the simulated data shows no clear patterns (see Figure 7). Although the clustering metric seems to be sensitive to patterns of missing data, no clear clustering structure was found on the estimated correlations.

The heatmap based on the multiplication data set (see Figure 8) shows clear clusters of items with related content. First, replicating the results of Study 1, a large cluster of items that involve a times 10, 100 or 1000 operator was found (*times 100* items). As expected, the items in this cluster have a strong negative correlation with the items from multiplication tables 2 through 9 (*table* items). The *table* items are not clearly represented in a single cluster. However, based on the content of the items the clustering solutions seem clearly interpretable. For example, items 700×80, 3000×80 and 80×6000 are placed close to items 8×7 and 8×6. A third weaker cluster seems to be present that included items that involve larger, more complicated, calculations without any times 10, 100, or 1000 operator.

For the addition data set (see Figure 9) the cluster solution appears less prominent, but certain item clusters are present and can be interpreted. First, a cluster of addition items with relatively small solutions is present (add 2, 3, 4 or 5), which correlate negatively with items with large solutions that involve adding tens (e.g., 40+10 and 6+90). A clear third small cluster is present with items that involve adding zero. However, a large set of addition items cannot be clearly assigned to a cluster. This indicates that the violation of unidimensionality may be less severe than for for the addition data set than the multiplication data set.

To conclude, the different analytics show that *table* and *times 100* multiplication items are best described by two different skills. Furthermore, the results based on the networked model shows that local skills can be estimated that provide important additions to the global skills currently used in the Math Garden. The correlations between these local skills should be described by multiple clusters that could be interpreted based on the item content. Future follow-up analyses using cross-validation techniques can determine both the weights in the expected score formula and the optimal number of clusters.

## 5. Discussion

The idiographic approach is essential in uncovering the rich dynamics of cognitive development. This is important for fundamental research, but also of practical relevance, especially in the educational context. The idiographic approach, however, requires highly frequent, psychometrically solid measurements of an individual. Once such data are collected, analyzing these data to gain insight into developmental and learning processes poses new challenges. We presented a new method to collect intensive time series to study intraindividual cognitive development using an innovative application of computerized adaptive testing to online learning platforms that schools can use for their students daily or weekly practice of various subjects, including language and mathematics. We then showed promising possibilities to analyze such intraindividual data, all concerning the development of mathematical skills. We explored different learning analytics based on time-series data of responses of children to single items. The results of Study 1 show that learning patterns in mathematics are irregular, with both relapses to lower ability and sudden changes to improved ability. In Study 2, different analyses indicated that mathematical skills consist of both global and local skills and that learning one local skill is not necessarily linked to learning another.

The results add to the main findings that result from microgenetic research [4]. Microgenetic research shows that cognitive development differs considerably between children and that strategy-usage is highly variable within children when a problem is repeatedly presented close in time [43]. According to Siegler and Crowley [4] multiple strategies are available to an individual and new more advanced strategies are not consistently applied. Over time more advanced strategies will replace older less adequate strategies. Hence, developmental change is not sudden, from strategy A to strategy B, but characterized by continuous shifts in the distribution of use of multiple (in)correct solution strategies [46], which Siegler refers to as the overlapping waves theory [47]. Moreover, this variability in strategy-use is often found in mathematics learning [48,49]. For example, children start with mostly simple counting strategies [50,51] and after they gain experience these will be replaced with more complex strategies, such as repeated addition for solving single digit multiplication [38]. For example, Lemaire and Siegler [52] showed that children often progress to more frequent use of complex strategies, but at each time point children use a mixture of strategies. The analyses in Study 1 add to these insights on development in mathematical abilities and show intraindividual and interindividual differences in learning trajectories.

The results of Study 2 also support this view on development in mathematics. Although each game consists of items that belong to a clearly defined domain (e.g., multiplication), we found multiple indications of multidimensionality. This might be surprising at first sight as tests consisting of very similar items (e.g., 4×6 and 12×3) are expected to be unidimensional. However, the enormous size of this data set yields very high power to detect interpretable sources of multidimensionality. What we found is that global skills in, for example multiplication, are supported by local skills, which can be interpreted as strategies for tackling specific items. Such strategies can exist next to each other and can be employed when applicable. As Straatemeier ([7], p. 174) proposed, ideally ability estimates would be based on unidimensional small item clusters within large item banks. We expect that separate ability estimates for these clusters will provide detailed insights into students’ skills. This would provide a middle way between the current Math Garden approach based on one common scale and a full idiographic approach with a measurement scale per person.

The presented approach is very promising for the domain of intelligence. In the current system, games already exist that tap abilities that are related to intelligence, such as deductive reasoning and working memory. Hence, a platform to collect high frequent data on a regular basis, in an environment that is attractive for children, is available and has been shown successful. The presented techniques can be applied to such data as well. Intelligence is also often assumed to be a general ability supported by local skills, such as working memory or perception. The suggested analyses provide insights into the stability of intelligence, components of intelligence, and their interrelations, on an individual level. This way we would come closer to a full idiographic measurement approach.

## Footnotes

1 Given the current large number of Math Garden players item ratings converge within about a week.

2 For many results in this section, formal significance tests could be provided. However, we refrain from doing so because: (1) no clear a priori hypothesis could be formulated and (2) due to the large data sets very small and uninteresting results become significant in a null-hypothesis testing framework. For example, the 35% did significantly differ from the expected 36% according to a simple proportion test ($X^{2}$(1) = 58.60, *p* = 1.936 ×$10^{-14}$)

3 For the permutation test we used 5000 replications where in each replication all items were randomly distributed between two sets. For each set we calculated each player’s average slopes (i.e., mean learning speed) and the correlation between both of these averages values.

## Abbreviations

The following abbreviations are used in this manuscript:

IRT	Item response theory
CAT	Computer adaptive testing
